# Assessment of Biosecurity Status in Commercial Chicken Farms Found in Bishoftu Town, Oromia Regional State, Ethiopia

**DOI:** 10.1155/2021/5591932

**Published:** 2021-08-16

**Authors:** Abdulbari Ismael, Adem Abdella, Shihun Shimelis, Asamenew Tesfaye, Yimer Muktar

**Affiliations:** ^1^College of Veterinary Medicine, Haramaya University, Haramaya, P.O. Box 138, Dire Dawa, Ethiopia; ^2^National Animal Health Diagnostic and Investigation Center, P.O. Box 04, Sebeta, Ethiopia; ^3^School of Veterinary Medicine, Woldia University, P.O. Box 400, Woldia, Ethiopia

## Abstract

A survey was undertaken from December 2017 to April 2018 to assess the biosecurity status of 44 commercial chicken farms established in Bishoftu town, Ethiopia, by interviewing farm owners using a structured questionnaire. The obtained data were summarized using frequency tables and analyzed with Pearson's chi-square test and Fischer's exact value using Stata 14 statistical software. From the assessed chicken farms, 31 (70.45%) were located within 0–50 m from the main road, 39 (88.64%) situated 500 m from the nearest farms, and 27 (61.36%) placed within 0–20 m from the residential areas. Forty-one (93.18%) participants disclosed that their employees did not receive training on biosecurity. From the assessed chicken farms, 30 (68.18%) had fences, 40 (90.91%) had footbaths at their gates, 31 (70.45%) prohibited visitors entrance, and 39 (88.64%) did not exchange equipment with other farms. In addition, 26 (59.09%) farms were easily accessed by wild birds, each of 42 (95.45%) farms purchased day-old chicks and feed, and 40 (90.91%) shared trucks with other farms as well. Among the assessed farms, only 2 (4.55%) had signages to restrict people's access, 9 (20.45%) had isolation rooms for diseased chickens, 14 (31.82%) disposed of dead birds properly, and 10 (22.73%) kept various types of records. Occupation (Fischer's exact value = 8.40; *P*=0.019), previous training (Fischer's exact value = 4.40; *P*=0.044), source of the premises (*χ*^2^ = 5.50; *P*=0.019), and farm capacity (Fischer's exact value = 13.50; *P*=0.002) were found statistically significantly associated with the farm biosecurity status. The farm biosecurity status was found to be good in farms that were run by civil servants, had trained employees, are owned premises, and were of large and medium scales. In conclusion, the higher poor biosecurity status on chicken farms calls for the implementation of good biosecurity practices in each farm as well as the provision of training to the farm owners and their employees.

## 1. Introduction

Poultry production is a very important type of animal production [1]. Poultry are efficient in producing high-quality protein (meat and eggs) [2]. However, poultry diseases remain the principal causes of failure in poultry production [3]. A successful animal production, including poultry, requires the adoption of good biosecurity practices [4], which is the most effective and inexpensive disease control measure [5]. Biosecurity in the poultry refers to a set of practices and measures taken to limit, control, or prevent the introduction and dissemination of infectious diseases in the poultry premises and facilities [6, 7]. A biosecurity program uses a combination of physical barriers such as fences, mesh wire, and directed actions to prevent the introduction of or minimize the spread of infectious disease-causing agents including the use of footbaths, carwash deep, and disinfection of farm equipment [2]. The three components of biosecurity measures are isolation, traffic control, and sanitation [8]. Van Limbergen et al. [4] and Sasaki et al. [9] disclosed that biosecurity is classified into internal and external. Biosecurity consists of conceptual, structural, and operational frameworks [10]. The conceptual category includes: the location of farms; structural: covering the building design and facilities to protect against entry of wild birds and predators; and operational: covering the routine disinfection, sanitation, and work procedures those farm employees and visitors follow [11]. The performance of birds is influenced by the biosecurity measures of the farms [5].

Ethiopia has an estimated poultry population of about 56.53 million [12]. The poultry production is characterized by small scavenging flocks of local chicken and few farms in the commercial subsector with varying flock sizes [13]. The small- and medium-scale producers constitute most of the commercial poultry production in Ethiopia [14]. So far, there have been very few attempts on the assessment of biosecurity measures of commercial poultry farms [15, 16] and poultry markets [17] of Ethiopia. Haftom et al. [15] reported that out of 25 small-scale poultry farms, 12 (44%) did not employ all-in all-out practice, 14 (56%) disposed of dead birds by throwing, and 16 (64%) kept different age groups together. The existing evidences depicted failure to fully practicing biosecurity measures in the integrated and larger commercial-scale types while virtually no or minimal routine application of biosecurity measures in the small-scale poultry production system [18].

Poultry production is important in Ethiopia as poultry play a major role in poverty alleviation, nutrition, and food security [14]. In Ethiopia, the chicken production system is classified into small-, medium-, large-, and integrated large commercial-scale production systems [18]. Ethiopia has small-, medium-, and large-scale intensive broiler and layer farms located in and around Addis Ababa, Debre Zeit (the now Bishoftu), Modjo, and Adama [19, 20]. Most small-scale poultry farms are located around Debre Zeit town in the Oromia region and Addis Ababa. The main commercial poultry farms, including Elflora, Agro Industry, Genesis, and Alema, are located around Debre Zeit in Oromia [19].

In Ethiopia, the application of biosecurity measures is limited [19], and to date, there is no information on the biosecurity status of commercial chicken farms in Bishoftu town. Therefore, the objective of this study was to assess the biosecurity practices of commercial chicken farms and identify the predictors of good biosecurity status in commercial chicken farms in Bishoftu.

## 2. Materials and Methods

### 2.1. Study Area

**Figure 1:**
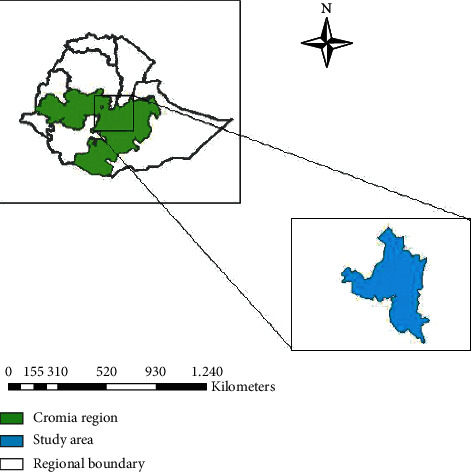
Map of Bishoftu town, East Shewa zone, Ethiopia. Source: Abunna et al. [23].

This study was carried out at Bishoftu town, East Shewa Zone of Oromia regional state. It is located 45 kilometers southeast of Addis Ababa, at 9°N latitude and 40°E longitude (Figure 1). Bishoftu town is located at an altitude of 1850 meters above sea level in the central high land of Ethiopia [21]. Farmers in the vicinity of Bishoftu use a mixed crop and livestock farming system. Moreover, Bishoftu and its surroundings have variable and yet representative agroecologies of the country. These agroclimatic zones are inhabited by different plant and animal species [22].

### 2.2. Study Population

The target population of the study comprised 50 commercial chicken farms at Bishoftu town that raise exotic breeds of chickens (predominantly Bovans and Lowmans) on a small scale (<1000 birds), medium scale (1000–10,000 birds), and large scale (>10,000 birds) [19]. These exotic chicken breeds are imported and are highly productive than the indigenous breeds of chicken.

### 2.3. Study Design and Sampling Technique

A cross-sectional population survey was carried out from December 2017 to April 2018 to evaluate the biosecurity status adopted by commercial chicken farms at Bishoftu town. The list of commercial chicken farms was obtained from the Ada'a Woreda Livestock and Fisheries Development Office. These farms were visited in person during data collection, and the owners were contacted and asked for their interest to participate in the biosecurity study. Verbal consent for participation was obtained only from 44 farms, while others refused to participate, and the reasons for refusal were not sought. The survey comprised face-to-face interviews of farm owners using a structured questionnaire. The interview was carried out by the same person.

### 2.4. Questionnaire Development

A structured questionnaire was developed and used to collect data on the biosecurity adopted by small-, medium-, and large-scale commercial chicken farms at Bishoftu. For the survey, owners' demography, farm characteristics, and relevant biosecurity practices were included in the questionnaire. Specific questions included were the demography of commercial chicken farm owners (gender, occupation, education level, experience in running chicken farms, and training received) and characteristics of the farms (premises, farm capacity, and farm type). Questions associated with the biosecurity assessment consisted of the conceptual framework such as distance from the main road, between farms, and from the residential area; presence of standing water; house type; housing position; and material used for house construction. In the structural framework, questions included were the presence of farm fence and gate, footbath, prohibition of vehicle entry, presence of tire bath/spray, prohibition of visitors, visitors sign-on logbook, no purchase of day-old chicken, no purchase of feed, no sharing of the truck with other farms, permanent rodent control, no access to stored fresh litter for wild birds, and presence of permanent wild bird control. At last, questions included in the operational biosecurity framework were the use of special cloth, footwear, masker, and hat; regular cleaning and disinfection; use of high-pressure sprayer; proper disposal of dead chicken; no other animals on the farm; veterinary consultation; in-between disinfection cycle; prophylactic treatment and vaccination; and so on. In general, a total of 69 closed questions were designed to obtain “yes” or “no” answers.

### 2.5. Data Collection

The questionnaire was pretested in ten chicken farms that were included in the survey, and care has been taken to avoid any misunderstanding or misinterpretation of the questions. The personal face-to-face interview was made to farm owners, managers, veterinarians, and employees. In addition, the farms were observed to assess the level of biosecurity at different levels.

### 2.6. Data Analysis

All collected data were entered into a Microsoft Excel spreadsheet, cleaned, and coded. Variables that are assumed to have a similar influence on the potential risk of introduction of contagious disease on the farm combined into a single variable, by producing a basic biosecurity score as a method described previously by [24]. The minimum and maximum biosecurity score obtainable on a farm were 0 and 72, respectively. The total sum assigned to the farm was divided by the maximum score that the farm could attain with the questions actually answered (72) and multiplying this proportion by 100 to obtain the percentage. A farm that gained >50% was considered having “good biosecurity practice”, and <50% as “poor biosecurity practice”. Statistical analyses were performed using STATA, version 13 statistical software. Pearson's chi-square or Fisher's exact test was used to estimate associations between demography of chicken farm owners and farm characteristics with the biosecurity status. A variable is said to have a significant effect when *P* < 0.05.

## 3. Results

### 3.1. Demography of Farm Owners

Of the 44 commercial chicken farm owners, 28 (63.64%) were males; 26 (59.09%) had higher education in various fields; 30 (68.18%) were traders; and 31 (70.45%) had previous experience in rearing chickens. Among those owners, 12 (27.27%) refused to disclose their level of education, and 20 (45.45%) did not receive training on chicken farm management. The demography of chicken farm owners is presented in Table 1.

### 3.2. Characteristics of Chicken Farms

As presented in Table 2, 32 (72.73%) were run on a rented premise, while only 12 (27.27%) were established on owned premises. The majority (86.36%) were categorized as small-scale chicken farms, and in 28 (63.64%) of them, only layers were reared.

### 3.3. Biosecurity Evaluation

#### 3.3.1. Conceptual Biosecurity

A total of eight biosecurity indicators were used to assess the concept of biosecurity and summarization revealed a mean score of 3.3 points with a standard deviation (SD) of 1.76. Among the 44 Bishoftu chicken farms, 31 (70.45%) were located within 0–50 m from the main road and 39 (88.64%) farms were established within 500 m from the nearest farm (Table 3). Furthermore, 27 (61.36%) were situated within 0–20 m from the residential area, and 10 (22.73%) were placed within 21–200 m. All (100%) the premises constructed for chickens were modified open sided with curtains, and 10 (22.73%) of them were built in the east-west direction. The employees of 41 (93.18%) farms disclosed that they did not receive any training on application biosecurity measures.

#### 3.3.2. Structural Biosecurity

For the evaluation of the structural biosecurity, 21 biosecurity measurements were considered. From chicken farms assessed, 30 (68.18%) had the fence; 40 (90.91%) had footbath at the gate; 31 (70.45%) prohibited entrance of visitors; 39 (88.64%) did not exchange equipment with other farms; each of 44 (100%) farms did not use surface water for drinking or cleaning; and 24 (54.55%) stayed informed regarding disease outbreak in the area (Table 4). However, only 6 (13.64%) undertook a permanent rodent control strategy. Furthermore, 26 (59.09%) farms were easily accessed by wild birds; each of 42 (95.45%) farms purchased day-old chicks and feed, and 40 (90.91%) shared trucks with other farms.

#### 3.3.3. Operational Biosecurity

As presented in Table 5, farmworkers of 15 (34.09%) farms did not wear special clothes; 36 (81.82%) farms did not use special footwear while operating on the farm; 31 (70.45%) did not undertake regular laundry to cap and overalls; and 27 (61.36%) farms did not store removed litter in a covered shed. However, only 2 (4.55%) had signage to restrict people's access; 9 (20.45%) had isolation rooms for diseased chickens; 14 (31.82%) farms properly disposed of dead birds; and 10 (22.73%) kept records.

#### 3.3.4. Overall Biosecurity Scores and Biosecurity Status

This study revealed the overall biosecurity scores for each farm. Thus, 11 (25%) farms got a score of >50%; therefore, their biosecurity practices were classed as “good”. The remaining 33 (75%) farms scored <50%; hence, their practices were graded as “poor”.

### 3.4. Assessment of Association between Biosecurity Level and Owners' Demography and Farm Characteristics

From the characteristics considered, occupation (Fisher's exact value = 8.40; *P*=0.019), previous training (Fisher's exact value = 4.40; *P*=0.044), source of the premises (*χ*^2^ = 5.50; *P*=0.019), and farm capacity (Fisher's exact value = 13.50; *P*=0.002) were found statistically significantly associated with the biosecurity level of the farm (Table 6).

From 24 farms whose owners received training, 9 (37.5) graded “good”, while 32 out of 38 small-scale and all broiler (*n* = 11) type chicken farms graded “poor”. Around 84.4% (27/32) of farms established on rented premises had poor biosecurity status. However, the association between owners' gender, education level, experience, and biosecurity status were statistically insignificant (*P* > 0.05; Table 6).

## 4. Discussion

This survey provides baseline information on the demography of people owning chicken farms and an insight into the biosecurity practices performed among the chicken farms established in Bishoftu town. However, limitations of this study were failure to assess the routine application and functionality and overlook the relative importance of each indicator to the overall biosecurity measures.

The majority of farm owners were males, completed higher education, and experienced in chicken rearing. Likewise, Ajewole and Akinwumi [25] and Kouam et al. [8] disclosed that the majority of small-scale broiler farmers were men. Kouam et al. [8] explained that this age-wise variation was attributed to the requirement of high commitment for success.

Although the majority of owners in this study had higher education, many did not receive training on biosecurity. Kouam et al. [8] linked this with the negligence of government officials to provide training in biosecurity as they lack understanding of the usefulness of biosecurity in animal husbandry, and this might also be partly the case in Bishoftu.

The majority of the chicken farms in Bishoftu town were small-scale farms with <5,000 birds rearing only layers on rented premises. In Egypt, 60% of broiler chicken farms were also small scale [6]. Many commercial chicken farms at Bishoftu were located near the main roads (<0–50 m) and in close proximity (<500 m). These present danger of airborne transmission of diseases from animals transported along the public road and between poultry farms. Thus, to minimize such transmission, the distance to the nearest poultry farm should be at least 500 m and preferably >1 km [26]. Several chicken farms at Bishoftu were located within 0–20 m from the residential area. This poses a biosecurity risk and considerable economic loss to the chicken farms as well as animal and public health problems through water/soil and air pollution [27].

In the present study, 30 out of 44 farms did not dispose of carcasses of dead chicken properly, and 27 out of 44 did not store removed litter at cover shade. However, carcasses of dead chicken and used litter must be disposed of properly because they are rich sources of infectious agents [4, 5]. Disposal of litter by spreading on nearby arable farmlands constitutes a risk of dissemination of disease-causing organisms [28]. Thus, those farms are at risk of the spread of infectious agents.

In this study, many farms were located far from standing water, and this finding varies from that of Kouam et al. [8] who reported that 73.5% of farms were located less than 500 m from a stream that poses a risk of pathogen transmission among poultry as water spots such as ponds, lakes, and rivers are attractive to migratory birds [29]. With regard to structural biosecurity, nearly all farms in this study had footbath at the gate, and this was higher than the finding of [30, 31] who reported 80% of the farms in Mekelle had footbath at the farm gate and only a small number of respondents set up a footbath at the farm entrance (37% from broiler farms and 18% from the layer farm). This difference may be due to better awareness of farm owners about disease spread by shoes of visitors and farmworkers. However, only a few poultry farms prohibited vehicle entry and apply tire spray/bath that poses a great risk as these trucks can spread a pathogenic microorganism onto the farm. Vehicle movement between farms is associated with farm infection [32].

The current study revealed that almost all farms purchase replacement chicken. This finding is higher than that of [33] who disclosed that 63.6% (7/11) of farmers sourced their birds from distributors without knowing the hatchery. Besides, the possibility of infection at the hatchery, day-old chicks may also be carriers of vertically transmitted (from hen to chick) pathogens such as *Mycoplasma* spp. [29]. This posed a substantial risk for poultry farms; even though the farm implements biosecurity measures upon the introduction of day-old chicken from other farms, the probability of pathogen entering the farm is high. This is because each poultry farm has its own risk profile for the introduction of pathogens, disease development, and spread of pathogens to other poultry farms [34]. Several studies have already pointed out that buying animals from different farms entails a greater risk of the introduction of disease-causing agents [35].

Many poultry farms at Bishoftu were prohibiting visitor's entry. This finding was in line with the study conducted in Egypt by Mohammed and Helal [36] who found 28.6% of small commercial poultry producers allowed visitors to enter poultry shades. But this finding disagrees with the finding of Birhanu et al. [31] who reported 76% of farms allowed visitors entry. The higher prohibition of visitors entrance observed in this study may be attributed to commercial poultry producers in Bishoftu town who have a better awareness of the risk of allowing visitors onto farms than those in Mekelle. Human movement among farms was shown to be an important risk factor for poultry diseases such as avian influenza [28] that is encouraging as visitors could access different farms and thereby introducing pathogens onto farms. To limit the risk of human movement, an entrance to a farm should be limited to one [37], and visitors should sign on a logbook when visiting a farm to enable rapid identification of people and farms during an outbreak [38]. Almost all of the farms involved in the present study bought feed from different sources that present the risk of introducing pathogens onto the farms. Besides lorries that can act as a mechanical vector, the feed can also be a source of infection. The feed can be contaminated with, for example, *Salmonella* spp., *Escherichia coli*, *Clostridium* spp., *Aspergillus* spp., and mycotoxins. The contamination of the feed can occur at different times during the production, storage, or transport [29].

From the surveyed farms only, a few farms implemented permanent rodent control, and wild birds were denied access to poultry houses. Failure to implement this practice constitutes a biosecurity risk as wild birds and rodents are carriers of pathogenic microorganisms that substantially affect the commercial poultry producers; especially, migratory wild birds are the cause of transboundary disease transmission. This finding was not in line with the study conducted in Khartoum that revealed that 33 (73.3%) of the farms controlled the entry of wild birds, rodents, or insects into poultry sheds or had strict measures to keep other poultry and domestic animals away from their flock [39]. The presence of reservoir wild birds influences the risk of introduction of poultry diseases such as avian influenza [37, 40, 41]. To attain biosecurity, rodents' entry must be minimum [5]. In operational BM perspectives, the present survey revealed that in more than half of the farms, regular cleaning and disinfection were undertaken, and this finding was lower from the study performed at Mekelle in which 88% of the farms assessed carry out regular cleaning and disinfection of equipment. Lower levels of biosecurity are associated with a higher prevalence and outbreak of avian disease [4, 42].

## 5. Conclusion

The findings from this study suggest that the practice of biosecurity implementation in commercial chicken farms at Bishoftu town was poor or lower with few farm owners who had been trained on the importance of proper biosecurity adoption. The majority of the biosecurity risks for chicken farms originated from inappropriate site selection, purchase of replacement day-old chicken and feed sources, and lack of training to farm employees. Therefore, there is a need to develop a biosecurity plan and find appropriate ways to educate the farm owners as well as farm employees and convince them to heed the plan.

## Figures and Tables

**Table 1:** The demography of chicken farm owners involved in biosecurity evaluation.

Farm owners demography	Category	Number of owners	Percentage (%)
Farm ownership	Female	10	22.72
	Male	28	63.64
	Both female and male	6	13.64

Owner's educational level	Primary and secondary education	6	13.64
	Higher education	26	59.09
	Not disclosed	12	27.27

Primary occupation	Trader	30	68.18
	Civil servant	8	18.18
	Others	6	13.64

Previous experience in rearing commercial chickens	Yes	31	70.45
	No	13	29.55

Previous training in biosecurity	Yes	24	54.55
	No	20	45.45

**Table 2:** The frequency and percentage of chicken farms by various farm characteristics.

Characteristics	Category	Number of farms	Percentage (%)
Sources of premises	Owned	12	27.27
	Rented	32	72.73

Farm capacity	Small scale	38	86.36
	Medium scale	3	6.82
	Large scale	3	6.82

Farm type	Layer	28	63.64
	Broiler	11	25.00
	Both layer and broilers	5	11.36

**Table 3:** The frequency and percentage of indicators of conceptual biosecurity.

Biosecurity indicators	Category	Number of farms	Percentage (%)
Distance of the farm from the main road (m)	0–50	31	70.45
	>50–100	4	9.09
	>100–300	4	9.09
	>300	5	11.36

Distance from the nearest farm (m)	<500	39	88.64
	≥500	5	11.36

Distance from the residential place (m)	0–20	27	61.36
	>20–200	10	22.73
	>200	7	15.91

No standing water near the farm	Yes	13	29.55
	No	31	70.45

Premise with modified open side and curtains	Yes	44	100
	No	0	0.00

Housing position	East-west	10	22.73
	Others	34	77.27

Chicken houses and hatcheries constructed of impervious material	Yes	19	48.18
	No	25	51.82

Biosecurity training to employee	Yes	3	6.82
	No	41	93.18

**Table 4:** The frequency and percentage of structural biosecurity indicators.

Biosecurity indicators	Yes (%)	No (%)
Presence of fence and gate	30 (68.18)	14 (31.82)
Presence of functional footbath	40 (90.91)	4 (9.09)
Prohibition of vehicle entry	17 (38.64)	27 (61.36)
Farm vehicle parked off the farm	10 (22.73)	34 (77.27)
Presence of only one vehicle entry point	26 (59.09)	18 (40.91)
Presence of tire bath/spray at the gate	4 (9.09)	40 (90.91)
Prohibition of entry of visitors	31 (70.45)	13 (29.55)
Visitors sign on logbook	1 (2.27)	43 (97.73)
No purchase of day-old chicken	2 (4.55)	42 (95.45)
No purchase of feed	2 (4.55)	42 (95.45)
No equipment exchange with other farms	39 (88.64)	5 (11.36)
No sharing of truck with other farms	4 (9.09)	40 (90.91)
No pet animal present in the farm	24 (54.55)	20 (45.45)
Presence of permanent rodent control	6 (13.64)	38 (86.36)
Presence of permanent wild bird control	3 (6.82)	41 (93.18)
Chicken area not accessible to wild bird	18 (40.91)	26 (59.09)
No access to stored fresh litter for wild birds	26 (59.09)	18 (40.91)
No access to stored food for wild bird	25 (56.82)	19 (43.18)
No feeding of chicken outside and no access to feed for wild birds	37 (84.09)	7 (15.91
Stay informed regarding the outbreak of poultry disease in the area	24 (54.55)	20 (45.45)
Surface water not used for drinking of chicken	44 (100)	0 (0.00)
Surface water not used for cleaning	44 (100)	0 (0.00)

**Table 5:** The frequency and percentage of operational biosecurity indicators.

Biosecurity indicators	Yes (%)	No (%)
Use of special cloth	29 (65.91)	15 (34.09)
Use of special footwear	36 (81.82)	8 (18.18)
Use of special masker	8 (18.18)	36 (81.82)
Use of special hat	8 (18.18)	36 (81.82)
Shower in and out	8 (18.18)	36 (81.82)
Regular laundering to cape and coveralls	13 (29.55)	31 (70.45)
No access to poultry compartment for visitors	34 (77.27)	10 (22.73)
Visitors special cloth	2 (4.55)	42 (95.45)
Visitors special footwear	8 (18.18)	36 (81.82)
Signage *t* the farm	2 (4.55)	42 (95.45)
Not keeping multiple ages together	40 (90.91)	4 (9.09)
Extending care from youngest to oldest	41 (93.18)	3 (6.82)
Employee not care for different age groups	3 (6.82)	41 (93.18)
Partial depopulation	24 (54.55)	20 (45.45)
Presence of paved places of discharge	22 (50.00)	22 (50.00)
Regular cleaning and disinfection	25 (56.82)	19 (43.18)
Used cleaning water is not drained outside	23 (52.27)	21 (47.73)
High pressure sprayer used for cleaning	21 (47.73)	23 (52.27)
Farm driver not permitted to enter poultry house	28 (63.64)	16 (36.36)
Staff not having contact with other farms	16 (36.36)	28 (63.64)
Dedicated worker to each chicken house	36 (81.82)	8 (18.18)
Proper disposal of dead chickens	14 (31.82)	30 (68.18)
Removed litter stored at cover shade	17 (38.64)	27 (61.36)
Applying insecticide on top of new litter	44 (100.00)	0 (0.00)
Two weeks of opening period after disinfection	32 (72.73)	12 (27.27)
No contact between poultry and other farm	37 (84.09)	7 (15.91)
No other farm animals in the farm	29 (65.91)	15 (34.09)
No poultry for hobby	41 (93.18)	3 (6.82)
Cleaning spilled feeds immediately	11 (25.00)	33 (75.00)
No access to stored food for rodents	16 (36.36)	28 (63.64)
Presence of isolation room for diseased chicken	9 (20.45)	35 (7.55)
Sick birds are regularly examined	16 (36.36)	28 (63.64)
Making a call to veterinarian when chicken appeared sick	34 (77.27)	10 (22.73)
Regular sero-monitoring	1 (2.27)	43 (97.73)
Vaccinating chickens for diseases known in the past	40 (90.91)	4 (9.09)
Vaccinating chickens according to the manufacturer's instruction	33 (75.00)	11 (25.00)
Using antibiotics only when birds are sick	29 (65.91)	15 (34.09)
Using antibiotics according to the recommended dosage	30 (68.18)	14 (31.82)
Not using expired vaccines/drugs	35 (79.55)	9 (20.45)
Presence of record-keeping	10 (22.73)	34 (77.27)

**Table 6:** Association between biosecurity level and owner's demography and farm characteristics.

Variable	Categories	Number of farms	Biosecurity status		Chi-square or Fisher's exact value	*P* value
			Good	Poor		
Farm ownership	Female only	10	2 (20.0)	8 (80.0)	0.55^a^	0.889
	Male only	28	8 (28.6)	20 (71.4)		
	Both male and female	6	1 (16.7)	5 (83.3)		

Owner's educational level	Elementary and high school	6	1 (16.7)	5 (83.3)	0.27^a^	1.000
	Higher education	26	7 (26.9)	19 (73.1)		
	Not disclosed	12	3 (25.0)	9 (75.0)		

Occupation	Trader^†^	30	6 (20.0)	24 (80.0)	8.40^a^	0.019^*∗*^
	Civil servant	8	5 (62.5)	3 (37.5)		
	Others	6	0 (0.0)	6 (100.0)		

Experience in rearing chicken	Yes	31	7 (22.6)	24 (77.4)	0.33^a^	0.706
	No	13	4 (30.8)	9 (69.2)		

Previous training on biosecurity	Yes	24	9 (37.5)	15 (62.5)	4.40^a^	0.044^*∗*^
	No					

Sources of poultry premises	Owned	12	6 (50.0)	6 (50.0)		
	Rented	32	5 (15.6)	27 (84.4)	5.50^b^	0.019^*∗*^

Farm capacity	Small scale	38	6 (15.8)	32 (84.2)	13.50^a^	0.002^*∗*^
	Medium scale	3	2 (66.7)	1 (33.3)		
	Large scale	3	3 (100.0)	0 (0.0)		

Farm type	Broilers only	11	0 (0.0)	11 (100.0)	5.03^a^	
	Layers only	28	9 (32.1)	19 (67.9)		
	Both broilers and layers	5	2 (40.0)	3 (60.0)		

^†^Businesses not linked with chicken, ^a^Fisher's exact value, ^b^Pearson's chi-square value, and ^*∗*^ significant (*P* < 0.05).

## Data Availability

The data are included in the tables within the manuscript.

## Supplementary Materials

Supplementary Materials A questionnaire format that was presented to chicken owners at Bishoftu to assess Biosecurity is given. Figure 1: representative map showing the country and study area where this research was conducted.
