# Supplementary material for: Assessment of Biosecurity Status in Commercial Chicken Farms Found in Bishoftu Town, Oromia Regional State, Ethiopia
Source: Vet Med Int. 2021 Aug 16;2021:5591932. doi: 10.1155/2021/5591932 (PMC8384549; doi:10.1155/2021/5591932)
Supplement: Supplementary Materials — A questionnaire format that was presented to chicken owners at Bishoftu to assess Biosecurity is given. Figure 1: representative map showing the country and study area where this research was conducted. [file 5591932.f1.pdf]

Questionnaire presented to chicken owners in Bishoftu town, Oromia Regional State, Ethiopia for the assessment of biosecurity status

---

Dear participant, at first we would like to acknowledge you for your consent to participate in this study aimed at evaluating the biosecurity status of your chicken farm. The findings of this result will help you to recognize the status of your farm in relation to the entry and spread of chicken diseases and seek measures to improve if in case there are pitfalls. No one will have access to the information gathered and it will be stored double secured in a computer.

**Part I. General Information on owners demography and farm characteristics**		
1. Farm identification:	Farm name _______________	Chicken breed(s)_________
2. Name of farm owner:	__________________________	
3. Farm ownership	☐ Female ☐ Male	☐ Both Female and male
4. Owner's education level	☐ Elementary and Secondary education	☐ Higher education
	☐ Not disclosed	
5. Primary occupation	☐ Trader ☐ Civil servant	☐ Others
6. Experience in rearing chicken	☐ Yes ☐ No	
7. Previous training on biosecurity	☐ Yes ☐ No	
8. Farm capacity	☐ Small-scale ☐ Medium-scale	☐ Largel-scale
9. Sources of poultry premises	☐ Owned ☐ Rented	
10. Farm type	☐ Layers ☐ Broilers	☐ Bother layers and broilers
**Part II. Biosecurity**		
**A) Conceptual biosecurity**		
1. How many is the distance of the farm from the main road (m) ?	☐ 0 – 50 ☐ > 50 – 100 ☐ > 300	☐ > 100 – 300
2. How many is the distance of the farm from the nearest farm (m)?	☐ < 500 ☐ $\geq$ 500	

3. How many is the distance of the farm from the residential place (m)?	☐ 0 – 20 ☐ >20 – 200	
4. Is there no standing water In the farm?	☐ Yes ☐ No.	
5. Is the premise built with modified open side and curtains?	☐ Yes ☐ No	
6. To which direction is the house oriented or positioned?	☐ East-West ☐ Others	
7. Are chicken house and hatcheries constructed of impervious material?	☐ Yes ☐ No	
8. Did employees of the farm receive training on biosecurity?	☐ Yes ☐ No	
**B) Structural biosecurity**		
1. Is there fence and gate in the farm?	☐ Yes ☐ No	
2. Is there footbath at the farm gate?	☐ Yes ☐ No	
3. Do you prohibit vehicle entry?	☐ Yes ☐ No	
4. Do farm vehicle parked off the farm?	☐ Yes ☐ No	
5. Is there only one vehicle entry point?	☐ Yes ☐ No	
6. Is tire bath/spray at the gate?	☐ Yes ☐ No	
7. Do you prohibit visitors from entering the farm?	☐ Yes ☐ No	
8. Do visitors sign on logbook?	☐ Yes ☐ No	
9. Is there **no** purchase of day-old chicken?	☐ Yes ☐ No	
10. Is there **no** purchase of chicken feed?	☐ Yes ☐ No	
11. Is there **no** equipment exchange with other farms?	☐ Yes ☐ No	

12. Is there **no** sharing of truck with others?	☐ Yes	☐ No	
13. Is surface water **not** used for drinking?	☐ Yes	☐ No	
14. Is surface water **not** used for cleaning?			
15. Is there **no** pet animal in the farm?	☐ Yes	☐ No	
16. Is there permanent rodent control in the farm?	☐ Yes	☐ No	
17. Is poultry area **not** accessible to wild bird?	☐ Yes	☐ No	
18. Do wild bird has **no** access to stored food?	☐ Yes	☐ No	
19. Is there **no** feeding outside and **no** access to the feed by wild birds?	☐ Yes	☐ No	
20. Do wild bird has **no** access to stored fresh litter?	☐ Yes	☐ No	
21. Is there permanent wild bird control?	☐ Yes	☐ No	
22. Do you stay informed regarding the disease outbreak in the area?	☐ Yes	☐ No	
**C) Operational biosecurity**			
1. Do farm workers use special cloth?	☐ Yes	☐ No	
2. Do farm workers use special footwear?	☐ Yes	☐ No	
3. Do farm workers use special masker?	☐ Yes	☐ No	
4. Do farm workers use special hat?	☐ Yes	☐ No	
5. Do farm workers shower when in and out?	☐ Yes	☐ No	
6. Is there regular laundering to hat and coveralls?	☐ Yes	☐ No	
7. Is there signage at the farm?	☐ Yes	☐ No	

8. Do visitors have **no** access to poultry compartment?	☐ Yes	☐ No	
9. Do visitors wear special cloth?	☐ Yes	☐ No	
10. Do visitors wear special footwear?	☐ Yes	☐ No	
11. Are there dedicated farm workers to each chicken house?	☐ Yes	☐ No	
12. Are multiple age-group **not** kept together?	☐ Yes	☐ No	
13. Is the order of work from the youngest to the oldest	☐ Yes	☐ No	
14. Do employee of the farm do**n't** care for different age-group?	☐ Yes	☐ No	
15. Is there partial depopulation of chicken?	☐ Yes	☐ No	
16. Is there regular cleaning and disinfection of premises?	☐ Yes	☐ No	
17. Are there paved places of discharge?	☐ Yes	☐ No	
18. Is there proper disposal of dead birds?	☐ Yes	☐ No	
19. Do farm workers have **no** contact with other farm?	☐ Yes	☐ No	
20. Is there isolation room for diseased chicken?	☐ Yes	☐ No	
21. Is used cleaning water **not** drained outside the farm?	☐ Yes	☐ No	
22. Is farm driver **not** permitted to enter poultry house?	☐ Yes	☐ No	
23. Is removed litter stored at cover shed?	☐ Yes	☐ No	
24. Do you use high pressure sprayer for cleaning?	☐ Yes	☐ No	
25. Do you allow two weeks of opening period after disinfection?	☐ Yes	☐ No	
26. Do you apply insecticide on to top of new litter?	☐ Yes	☐ No	

27. Do you apply insecticide on top of new litter?	☐ Yes	☐ No	
28. Are there other farm animals in the farm?	☐ Yes	☐ No	
29. Are there **no** any poultry for hobby?	☐ Yes	☐ No	
30. Is stored feed **not** accessible to rodents?	☐ Yes	☐ No	
31. Do you call veterinarians when a chicken appear sick?	☐ Yes	☐ No	
32. Do sick birds get examined regularly?	☐ Yes	☐ No	
33. Is there regular seromonitoring of chickens?	☐ Yes	☐ No	
34. Are chicken vaccinated only for known disease in the past?	☐ Yes	☐ No	
35. Do you vaccinate chicken only according to the manufacturer's instruction?	☐ Yes	☐ No	
36. Do you use antibiotics when only birds are sick?	☐ Yes	☐ No	
37. Do you use antibiotics based on the recommended dosage?	☐ Yes	☐ No	
38. **Don't** you use expired vaccines or drugs?	☐ Yes	☐ No	
39. Do you keep records in the farm?	☐ Yes	☐ No	
40. Is spilled feed cleaned up immediately?	☐ Yes	☐ No	
